# Integrated transcriptomic and machine learning analysis reveals novel diagnostic biomarkers for adolescent major depressive disorder

**DOI:** 10.3389/fpsyt.2026.1712225

**Published:** 2026-01-30

**Authors:** Runxu Yang, Linling Jiang, Junxi Pan, Kun Lian, Yilin Xie, Yiqing He, Ziyang Huang, Qingqing Qi, Jin Lu

**Affiliations:** 1 Psychiatric Department, First Affiliated Hospital of Kunming Medical University, Kunming, China; 2 Mental Health Institute of Yunnan, First Affiliated Hospital of Kunming Medical University, Kunming, China; 3 Yunnan Clinical Research Center for Mental Health, Kunming, China; 4 Yunnan Clinical Center for Mental Health, Kunming, China; 5 Department of Clinical Laboratory, First Affiliated Hospital of Kunming Medical University & Yunnan Province Clinical Research Center for Laboratory Medicine, Kunming, China; 6 Department of Neurosurgery, Second Affiliated Hospital of Kunming Medical University, Kunming, China

**Keywords:** adolescent major depressive disorder, transcriptomics, biomarkers, peripheral blood mononuclear cells, machine learning, immune-metabolic dysregulation

## Abstract

**Introduction:**

The lack of objective biomarkers and mechanistic understanding of adolescent Major Depressive Disorder (MDD) impedes early diagnosis and targeted intervention.

**Methods:**

To elucidate peripheral molecular biomarkers for adolescent MDD, we performed RNA sequencing on peripheral blood mononuclear cells (PBMCs) from 15 adolescents with MDD and 15 age- and sex-matched healthy controls. Differential expression analysis and protein-protein interaction (PPI) network construction were utilized to identify key regulatory genes. The expression of core targets was validated using RT-qPCR and ELISA. To establish a robust diagnostic model, an integrated feature selection strategy combining Least Absolute Shrinkage and Selection Operator (LASSO), Support Vector Machine-Recursive Feature Elimination (SVM-RFE), and Random Forest algorithms was applied to screen candidate biomarkers.

**Results:**

Transcriptomic profiling identified 367 differentially expressed genes characterized by a dual signature of innate immune activation and compensatory hypoxic responses. Eight core hub genes were identified and experimentally validated, revealing a dichotomous expression pattern: upregulation of erythroid-related and inflammatory factors (SLC4A1, HBB, GYPA, IL6) and downregulation of neurotrophic and remodeling factors (IGF1, CSF2, MMP9, CXCR1). Notably, lower expression levels of MMP9 and CXCR1 were significantly correlated with higher Hamilton Depression Rating Scale (HAMD) scores, indicating greater symptom severity. The multi-algorithm machine learning approach identified a consensus three-gene diagnostic panel comprising SLC4A1, IGF1, and MMP9, which achieved a high classification accuracy with an Area Under the Curve (AUC) of 0.867.

**Conclusion:**

This study delineates a systemic molecular landscape of adolescent MDD defined by the coexistence of hypoxic compensation and neurotrophic/remodeling failure. The identified three-gene biosignature (SLC4A1, IGF1, MMP9) offers a promising, objective tool for the early diagnosis of adolescent depression, highlighting the immune-metabolic interface as a critical avenue for future precision medicine.

## 1 Introduction

MDD is a widespread psychiatric condition characterized by persistent depressed mood, anhedonia, and cognitive impairment, substantially compromising social functioning and quality of life (1, 2). As reported by the World Health Organization (WHO), more than 300 million people are affected by MDD globally, with increasing incidence and recurrence rates exacerbating the burden on healthcare and economic systems worldwide (3, 4).

Despite extensive investigation, the precise molecular underpinnings of MDD remain elusive, impeding early diagnosis and effective management. Emerging evidence implicates dysregulation of neurotransmitter systems (5, 6), inflammatory activation (7–10), and impaired neuroplasticity (11, 12) as pivotal contributors to its pathogenesis. However, these mechanistic insights, while foundational, have not yet translated into reliable biomarkers, underscoring the need for more comprehensive, system-level approaches.

Advances in transcriptomic profiling via RNA sequencing (RNA-seq) (13) have enabled systematic exploration of the molecular architecture of psychiatric diseases. RNA-seq facilitates genome-wide identification of differentially expressed genes (DEGs) in pathological states, offering insights into the involved biological pathways and regulatory networks. This methodology is particularly advantageous for detecting peripheral biomarkers, as blood-derived transcripts can reflect systemic and central nervous system (CNS) alterations in a minimally invasive manner.

Prior transcriptomic studies conducted primarily in adults, mostly using PBMCs, have yielded important insights, revealing immune dysregulation and peripheral inflammation, including elevated pro-inflammatory mediators such as IL-6 and MMP9 (14–16). However, the applicability of these adult-derived signatures to adolescents is highly uncertain. Adolescent MDD presents distinct etiological challenges, arising during a critical neurodevelopmental window shaped by dynamic hormonal changes, ongoing brain maturation, and unique psychosocial stressors. This developmental disparity is critical; molecules integral to growth and immune regulation, such as insulin-like growth factor-1 (IGF1) and granulocyte-macrophage colony-stimulating factor (CSF2), may exert unique roles in adolescent pathophysiology (17). Consequently, the scarcity of reliable, age-specific biomarkers for adolescent MDD hinders early identification and intervention, often resulting in chronicity and adverse long-term outcomes.

To address this critical gap, we conducted blood-based RNA sequencing in adolescents with MDD to characterize systemic molecular alterations. Specifically, we aimed to: 1) identify adolescent-specific differentially expressed genes (DEGs); 2) elucidate their functional implications via pathway enrichment analyses; and 3) determine disease associations to reveal broader systemic relevance. By delineating the unique molecular signatures of adolescent MDD, this work seeks to provide a more integrative understanding of the disorder, identify potential biomarkers for early diagnosis, and reveal novel therapeutic targets tailored to this vulnerable population.

## 2 Methods

### 2.1 Participants and procedure

This study recruited 15 adolescents (aged 12–17) with DSM-5–defined MDD and HAMD-17 scores ≥17 from the Department of Psychiatry, and 15 age- and sex-matched healthy controls from the Pediatric Health Center of the First Affiliated Hospital of Kunming Medical University between June and December 2024. All diagnoses were confirmed by attending psychiatrists. Written informed consent was obtained from participants and their legal guardians. Exclusion criteria included organic comorbidities, inflammatory or autoimmune diseases, schizophrenia spectrum disorders, bipolar disorder, and substance use disorders; for healthy controls, additional exclusions were any personal or family history of psychiatric illness. Clinical assessments were conducted by trained professionals using standardized procedures. All procedures were approved by the institutional review board of the First Affiliated Hospital of Kunming Medical University and conducted in accordance with the 2013 Declaration of Helsinki. Clinical and demographic characteristics are listed in **Table 1**.

**Table 1:** Demographic and clinical profiles of the MDD and HC groups.

Characteristic	MDD (n = 15)	HC (n = 15)	t/χ^2^/z	*p*-Value
Gender (male/female)	8/7	7/8	χ² = 0	1
Age (years) (Mean ± SD)	14.5 ± 1.7	14.7 ± 1.7	t = 0.43	0.670
HAMD-17 score (median/IQR)	27 (23.5-30.5)	3 (1.5-4.5)	z = 0	0.000

HC, healthy control; HAMD, Hamilton Depression Rating Scale; MDD, major depressive disorder.

### 2.2 PBMCs isolation

PBMCs were isolated from 2 mL of freshly collected whole blood diluted 1:1 with phosphate-buffered saline. The diluted sample was gently layered onto 3 mL of Ficoll–Paque PLUS and centrifuged at 3000 g for 20 min at 18–20°C. Following density gradient separation, the mononuclear cell layer was carefully aspirated and washed. Cells were then subjected to a low-speed spin (60–100g, 10min) to remove residual debris. After discarding the supernatant, cell pellets were lysed in TRIzol and stored at −80°C for subsequent analyses.

### 2.3 RNA extraction, library preparation and sequencing

Total RNA was isolated from monocytes using TRIzol reagent (MJZol total RNA extraction kit). RNA quality and integrity were evaluated by measuring the A260/A280 ratio with a NanoDrop ND-2000 (Thermo Scientific, USA) and determining RNA integrity numbers using an Agilent 5300 Bioanalyzer (Agilent Technologies, USA). Only samples that passed predefined quality control thresholds were advanced to library construction. Paired-end libraries were generated using the Illumina Stranded mRNA Prep Ligation (Illumina, USA) following the manufacturer’s instructions. Briefly, 1μg of total RNA was enriched for mRNA using oligo(dT) magnetic beads and subsequently fragmented in the first-strand synthesis buffer. First-strand cDNA was synthesized using random primers and reverse transcriptase, followed by second-strand synthesis with DNA polymerase I, RNase H, and dNTPs. The resulting double-stranded cDNA was end-repaired, adaptor-ligated, and PCR-amplified. Purified libraries were assessed for quality on an Agilent 5300 Bioanalyzer before sequencing on the Illumina NovaSeq Reagent Kit platform.

### 2.4 Data preprocessing

Raw image files were processed with llumina BCL Convert (version 3.9.3) to generate FASTQ-format reads. Quality control included adapter trimming and removal of low-quality sequences, yielding high-quality clean reads. Clean reads were aligned to the reference genome using hisat2 (version 2.2.1). Gene-level quantification was performed with RSEM (version 1.3.3), and transcript abundance was expressed as fragments per kilobase of transcript per million mapped reads (FPKM) to enable standardized comparison across samples.

### 2.5 Bioinformatics analysis and screening strategy

Differential expression analysis was conducted in a discovery framework, and nominal p-values (p < 0.05 and |Log_2_FC| > 2) were used for downstream network and validation analyses, using the edgeR R package (version 4.4.2). This criterion was designed to capture high-magnitude regulators that might be penalized by FDR correction due to inter-individual heterogeneity. Expression patterns were visualized using the edgeR R package (version 4.4.2) and pheatmap (version 1.0.13) R packages. Functional enrichment (GO pathways) was conducted via Metascape. The PPI network was mapped using the STRING database and visualized in Cytoscape. Key modules and hub genes were identified using the MCODE and CytoNCA plugins (Mediator Number Centered algorithm), respectively.

### 2.6 Quantitative real-time polymerase chain reaction

To ensure the technical reliability of the transcriptomic profiles, qPCR validation was performed using the identical discovery cohort (20 adolescents with MDD and 20 matched healthy controls). Total RNA was extracted from PBMCs using TRIzol reagent. To validate transcriptomic findings, the expression of selected DEGs was quantified by qPCR. Relative mRNA levels were calculated using the 2^−ΔΔCt^ method. The validation panel targeted the following genes: solute carrier family 4 member 1 (SLC4A1), hemoglobin subunit beta (HBB), insulin-like growth factor 1 (IGF1), colony stimulating factor 2 (CSF2), matrix metallopeptidase 9 (MMP9), C-X-C motif chemokine receptor 1 (CXCR1), interleukin 6 (IL6), and glycophorin A (GYPA). Primers were designed using the NCBI Primer-BLAST. The primers for the eight main core factors are listed in **Supplementary Table S1**.

### 2.7 Enzyme-linked immunosorbent assays

Enzyme-linked immunosorbent assay (ELISA) Serum levels of the proteins encoded by the validated hub genes—SLC4A1, HBB, IGF1, CSF2, MMP9, CXCR1, IL6, and GYPA—were quantified using commercial ELISA kits. All assays were performed strictly according to the manufacturers’ protocols. Detailed information regarding the specific kits, including manufacturers and catalog numbers, is provided in **Supplementary Table S2**.

### 2.8 Screening and identification of diagnostic biomarkers using machine learning

To identify robust diagnostic biomarkers for MDD and mitigate the risk of overfitting due to the limited sample size (N = 30), we employed an integrated feature selection strategy combining three distinct machine learning algorithms: LASSO, SVM-RFE), and Random Forest (RF). For LASSO regression, we implemented a Stratified Stability Selection approach using the glmnet R package (version 4.1.10). Unlike standard cross-validation, which can be unstable in small datasets, this method involved 100 bootstrap iterations. In each iteration, stratified sampling was performed to maintain a balanced ratio of case (MDD) to control samples (15 vs. 15). Genes that were selected in more than 60% of the iterations were considered stable features. Concurrently, the SVM-RFE algorithm was applied using the caret R package (version 7.0.1) to rank features based on their contribution to model accuracy. To ensure a rigorous evaluation, we utilized Leave-One-Out Cross-Validation (LOOCV), which is the optimal validation strategy for small sample sizes, to determine the feature subset that yielded the highest classification accuracy. Additionally, the Random Forest algorithm was utilized (randomForest package [version 4.7.1.2], ntree = 2000) to evaluate feature importance based on the Mean Decrease Gini index. Finally, the intersecting genes identified by all three algorithms were determined using a Venn diagram. These overlapping genes were defined as the final hub diagnostic markers. A logistic regression model was constructed using these markers, and its predictive performance was evaluated using Receiver Operating Characteristic (ROC) curve analysis.

### 2.9 Statistical analysis

Statistical analyses were conducted using the R computing environment (version 4.4.2). Continuous variables are expressed as mean and standard deviation (SD) for normally distributed data or median with interquartile range (IQR) for non-normally distributed data. Data normality was assessed using the Shapiro–Wilk test. For between-group comparisons, the independent two-sample t-test was employed for normally distributed continuous variables, whereas the Mann–Whitney U test was utilized for non-normally distributed data. Given the limited sample size (N = 30) and potential non-normal distribution of gene expression data, partial Spearman’s rank correlation analysis was performed to evaluate the relationship between hub gene expression and HAMD scores. Age and gender were included as covariates to control for potential confounding effects. The ppcor R package (version 1.1) was used for the analysis. All statistical tests were two-sided, and a p-value < 0.05 was considered statistically significant.

## 3 Results

### 3.1 Global transcriptomic profiling and functional characterization

**Figure 1:**
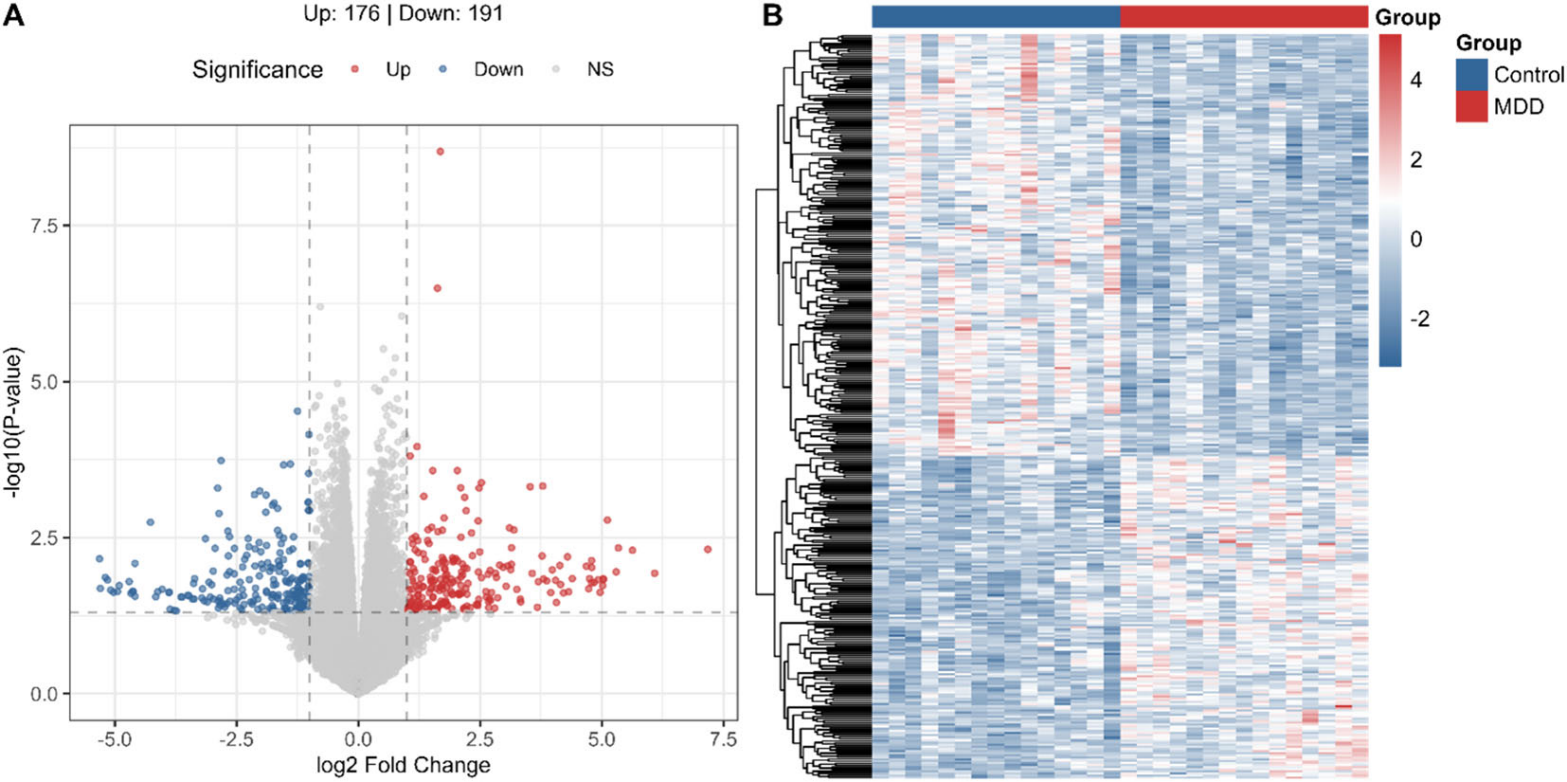
Differential gene expression in PBMCs from adolescents with MDD and healthy controls. **(A)** Overview of transcriptomic alterations and relative expression patterns in MDD compared with healthy controls. **(B)** Identification of 367 differentially expressed genes in PBMCs (p < 0.05, |log_2_FC| > 1), including upregulated genes (red) and downregulated genes (blue).

To systematically characterize the peripheral molecular signatures of adolescent depression, we performed RNA-sequencing on PBMCs from 15 adolescents with MDD (HAMD-17 scores: 19–33) and 15 age- and sex-matched healthy controls (HAMD-17 scores: 0–6). Differential expression analysis identified 367 dysregulated genes (p < 0.05, |Log_2_FC| > 1), comprising 176 upregulated and 191 downregulated transcripts in the MDD cohort (**Figures 1A, B**). Functional enrichment analysis uncovered a dual signature of systemic immune dysregulation and metabolic stress. Specifically, biological processes were predominantly enriched in innate immune responses, such as myeloid leukocyte and neutrophil activation. Concurrently, molecular functions converged on hemoglobin complex and oxygen carrier activities, suggesting a convergence on pathways related to immune activation and oxygen transport, which may reflect systemic metabolic stress in adolescent MDD (**Supplementary Figure S1**).

### 3.2 Topological analysis of PPI networks and experimental validation of hub genes

**Figure 2:**
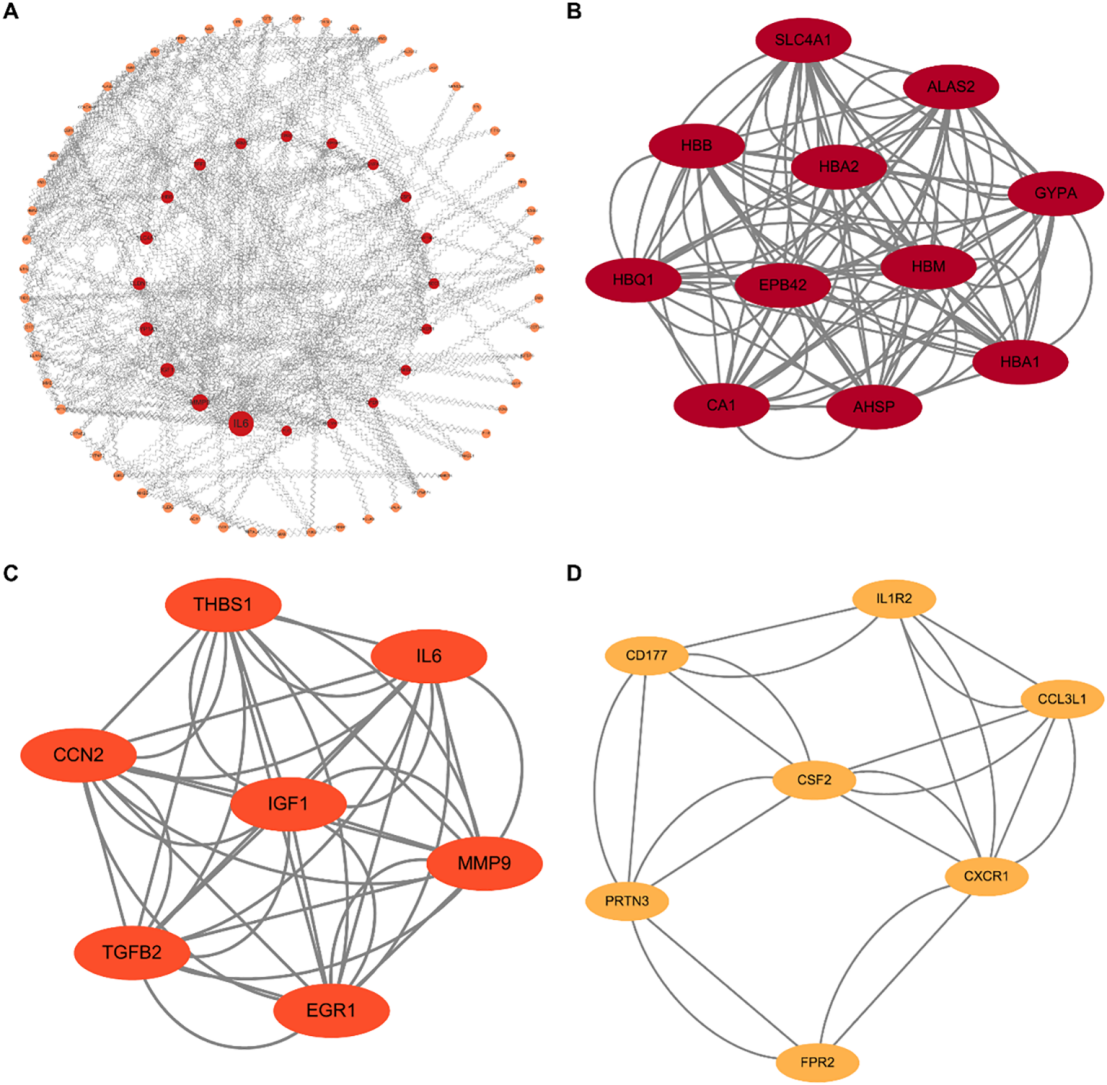
Topological analysis of PPI networks. **(A)** Overview of the global PPI network constructed from the differentially expressed genes (N = 367). Red nodes indicate upregulated genes, and orange nodes indicate downregulated genes. **(B–D)** Identification of key functional modules using the MCODE algorithm. **(B)** The erythroid-related module (e.g., *SLC4A1*, *HBB*, *GYPA*). **(C)** The growth factor and cytokine regulatory module (e.g., *IL6*, *IGF1*, *MMP9*). **(D)** The immune trafficking module (e.g., *CXCR1*, *CSF2*). These hub clusters reveal the core molecular machinery underlying the immuno-metabolic dysregulation in MDD.

To identify key regulatory nodes characterized by high connectivity and substantial magnitude of dysregulation, we constructed a PPI network based on the network discovery set (367 nominally significant DEGs). We employed a dual-algorithm strategy to ensure robustness: CytoNCA prioritized the top 25 nodes based on subgraph centrality, while MCODE detected highly interconnected functional modules. The intersection of these independent approaches converged on a consensus list of 8 core hub genes (**Figure 2**), comprising erythroid-related factors (SLC4A1, HBB, GYPA) and immune/growth regulators (IL-6, CSF2, MMP9, CXCR1, IGF1).

**Figure 3:**
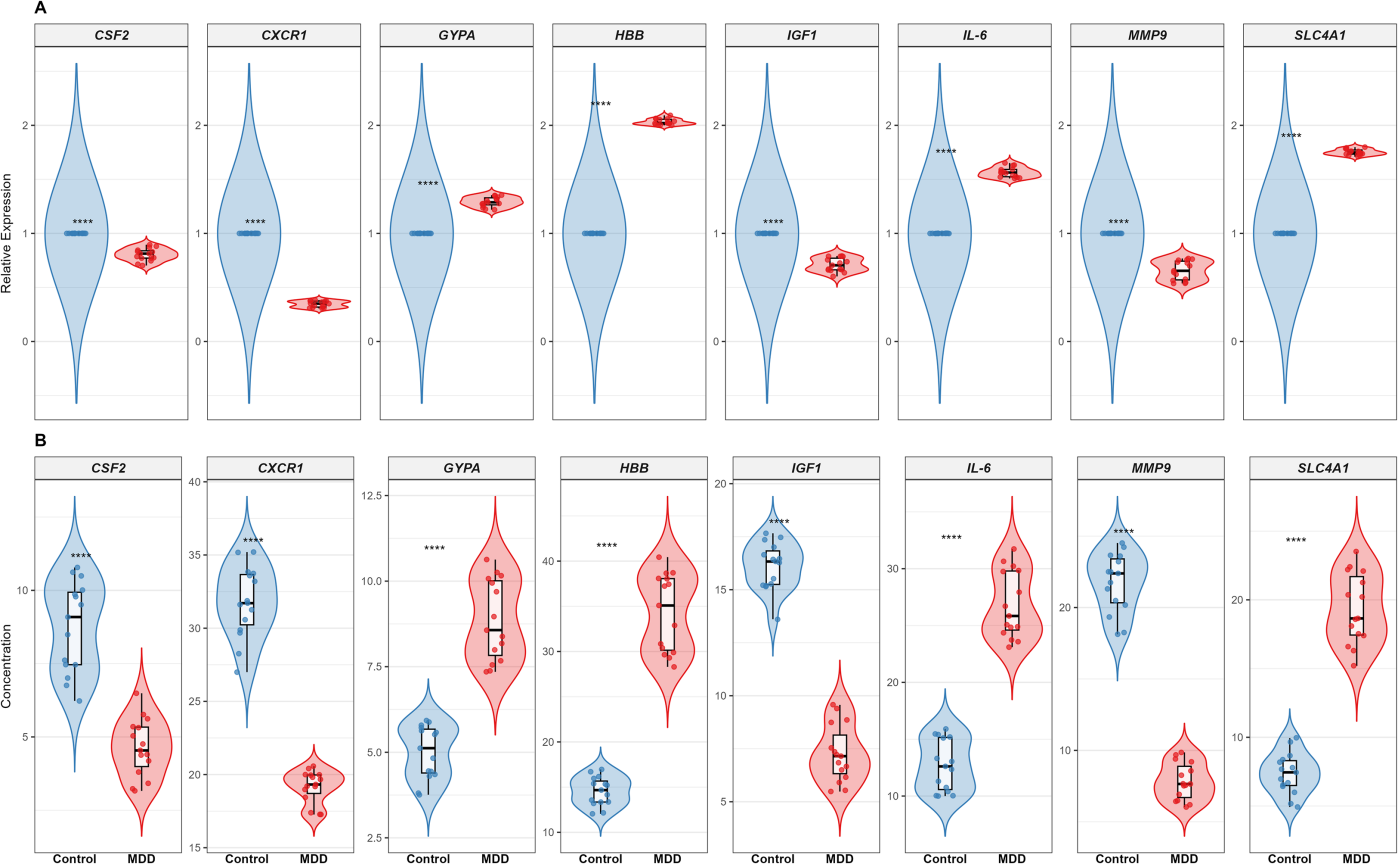
Experimental validation of core hub genes. **(A)** Validation of mRNA expression levels in PBMCs using RT-qPCR. **(B)** Validation of protein levels in plasma using ELISA. Data are presented as mean ± SEM. Statistical significance was determined by unpaired t-test (*p < 0.05, **p < 0.01, ***p < 0.001).

To validate these in silico findings, we performed cross-validation using RT-qPCR and ELISA. Both assays yielded consistent results, revealing a distinct dichotomous expression pattern: upregulated cluster: expression levels of SLC4A1, HBB, GYPA, and IL-6 were significantly elevated in the MDD group, suggesting a compensatory enhancement of oxygen-carrying capacity and erythropoiesis; downregulated cluster: conversely, IGF1, CSF2, MMP9, and CXCR1 exhibited significant downregulation, indicating a suppression of specific cell growth and inflammatory trafficking pathways. Collectively, these validation data confirm that the identified core hubs orchestrate a complex shift involving enhanced erythroid function and altered immune modulation (**Figure 3**).

### 3.3 Association of hub gene expression and protein levels with depressive symptom severity

**Figure 4:**
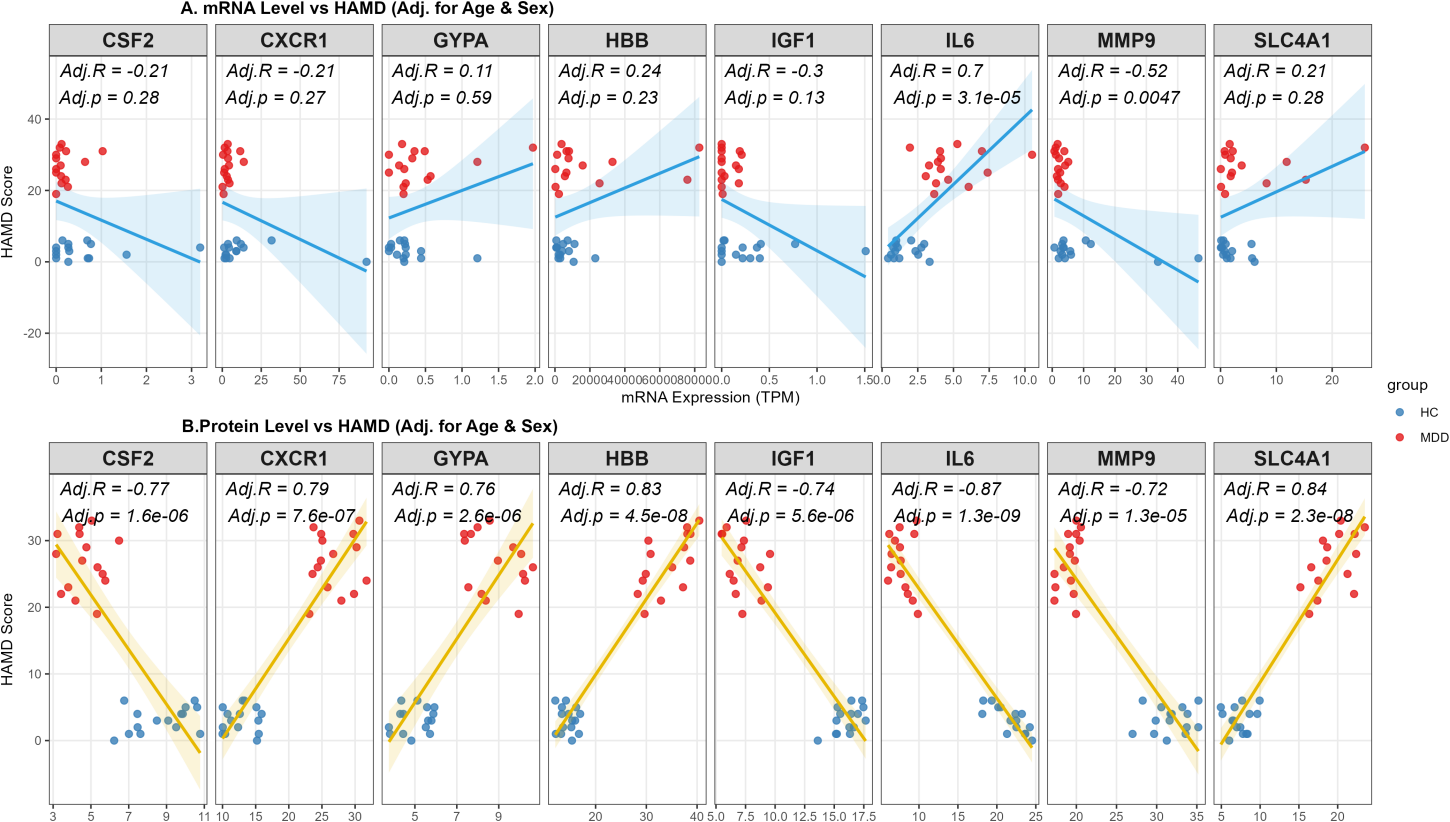
Associations between hub gene biomarkers and depression severity at proteomic and transcriptomic levels. **(A)** Partial Spearman’s rank correlation analysis between peripheral blood mRNA expression levels (RNA-seq) and HAMD scores. The regression line is shown in blue. **(B)** Partial Spearman’s rank correlation analysis between ELISA of 8 hub genes and HAMD scores. The regression line is shown in gold. Note: The analysis was performed in the entire study cohort (N = 30), including Healthy Controls (n=15) and patients with MDD (n=15). All correlation coefficients (Adj.R) and P-values (Adj.p) were adjusted for age and sex as covariates. The regression lines with 95% confidence intervals (shaded areas) indicate the trends of the associations. Blue dots represent HC subjects, and red dots represent MDD patients.

To investigate the clinical relevance of the identified hub genes, we assessed the associations between their expression levels and depressive symptom severity (HAMD scores) using partial Spearman correlation analyses adjusted for age and sex. At the transcriptomic level, significant associations were specific to IL-6 and MMP9 (P < 0.01) (**Figure 4A**). MMP9 showed a consistent negative correlation at both mRNA (Adj.r = -0.52) and protein levels (Adj.r = -0.72), indicating a convergent transcript–protein association with symptom severity. Intriguingly, IL-6 mRNA was positively correlated with symptom severity (Adj.r = 0.70), contrasting with its protein level. At the proteomic level, the analysis revealed broader consistency; all eight candidate proteins correlated significantly with HAMD scores (P < 0.05) (**Figure 4B**). HBB (Adj.r = 0.83, P < 0.001) and SLC4A1 (Adj.r = 0.83, P < 0.001) demonstrated robust positive associations, while IL-6 protein (Adj.r = -0.87, P < 0.001) was negatively correlated. The discordance between transcript and protein levels of IL-6 suggests post-transcriptional regulation, which is further discussed below.

### 3.4 Identification and validation of diagnostic biomarkers for MDD

**Figure 5:**
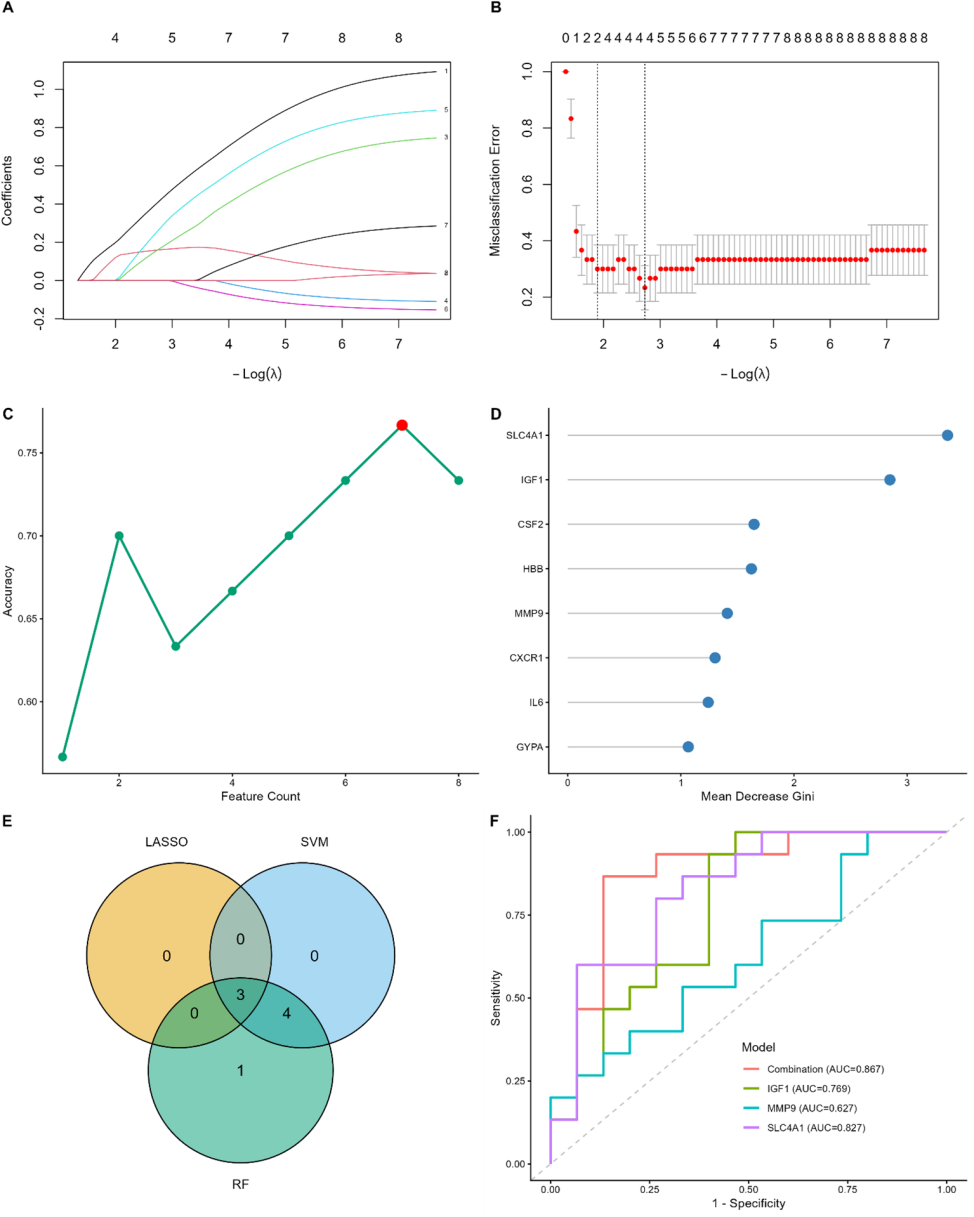
Screening and validation of diagnostic biomarkers for MDD based on machine learning. **(A, B)** LASSO regression analysis. **(A)** LASSO coefficient profiles of the candidate genes. **(B)** Selection of the optimal lambda parameter using LOOCV. **(C)** SVM-RFE algorithm for feature selection. The plot shows the accuracy changes with the number of features; the red dot indicates the maximum accuracy (0.90) with 7 genes. **(D)** Random Forest feature importance ranking. Genes are ranked by the Mean Decrease Gini index; the top genes contribute most to the classification. **(E)** Venn diagram showing the intersection of candidate genes identified by LASSO (n=3), SVM-RFE (n=7), and Random Forest (n=8). Three common genes were identified. **(F)** ROC curve analysis for the 3 hub genes (SLC4A1, IGF1, and MMP9). The AUC values indicate the diagnostic performance of each gene.

To identify robust diagnostic biomarkers for MDD within the limited sample size (N = 30), we implemented a multi-algorithmic machine learning approach integrating LASSO regression, SVM-RFE, and Random Forest based on the 8 candidate hub genes. First, LASSO stability selection with 100 stratified bootstrap iterations identified 3 stable genes with selection probabilities exceeding 0.6 (**Figures 5A, B**). Second, the SVM-RFE algorithm with leave-one-out cross-validation (LOOCV) achieved optimal classification accuracy with 7 features (**Figure 5C**). Third, the Random Forest model ranked the candidates based on the Gini index (**Figure 5D**). To ensure reliability, we intersected the candidate genes identified by these three independent methods. The Venn diagram revealed that 3 genes (SLC4A1, IGF1, and MMP9) were consistently selected (**Figure 5E**) and were thus retained as the final diagnostic biomarkers. This combined diagnostic model achieved an AUC of 0.867 (**Figure 5F**).

Subsequently, we validated these findings using ELISA data. Notably, SVM-RFE achieved a classification accuracy of 1.000 using IGF1 alone (**Supplementary Figure S3C**). Given this near-perfect discrimination within this cohort (AUC = 1.000), we conducted a permutation test with 1,000 iterations to rigorously assess the risk of overfitting. As shown in **Supplementary Figure S3F**, the observed AUC was significantly distinct from the random permutation distribution (P < 0.001).

## 4 Discussion

The absence of objective biological benchmarks for adolescent MDD remains a critical bottleneck in early identification and precision medicine. In the present study, we performed comprehensive transcriptomic profiling of PBMCs to unravel the peripheral molecular landscape underlying adolescent major depressive disorder (MDD). Our data revealed a distinctive dual-signature characterized by innate immune activation and may reflect a compensatory transcriptional response related to oxygen transport or metabolic stress. Through topological network analysis and experimental validation, we identified a consensus set of eight core hub genes. These genes exhibited a dichotomous expression pattern: an upregulation of pro-inflammatory and erythroid-related factors (IL6, SLC4A1, HBB, GYPA) contrasted with a suppression of neurotrophic and remodeling factors (IGF1, CSF2, MMP9, CXCR1). Furthermore, by integrating three independent machine learning algorithms, we successfully constructed a robust diagnostic model based on SLC4A1, IGF1, and MMP9, which demonstrated high accuracy (AUC = 0.867) in distinguishing adolescent MDD patients from healthy controls.

Functional enrichment analyses revealed that these molecular alterations converge on critical physiological axes, specifically immune activation (18) and inflammatory signaling (19), alongside metabolic and signal transduction perturbations (20–22). This suggests that the functional activation of immune cells, particularly neutrophils, plays a pivotal role in MDD. Recent studies have increasingly highlighted the link between MDD and a chronic low-grade inflammatory state (23–25). The activation of innate immune cells, such as neutrophils (26, 27) and macrophages (28–30), mediates neuroinflammatory responses through the release of inflammatory factors, which in turn affect central nervous system (CNS) function.

A salient and novel finding of our study is the coordinated upregulation of erythroid-related genes (SLC4A1, HBB, GYPA) in the PBMCs of MDD patients. Under physiological conditions, these genes are predominantly expressed in the erythroid lineage; their ectopic or elevated expression in peripheral mononuclear cells suggests a systemic response to metabolic stress or hypoxia. Emerging evidence links depression to mitochondrial dysfunction and oxidative stress (31–33), which may lead to a state of “pseudohypoxia” at the cellular level (34, 35). SLC4A1 (Band 3) functions as a critical regulator of systemic pH and CO2 transport. Its dysregulation in blood transcriptomics often reflects a compensatory response to chronic metabolic acidosis or oxidative stress, serving as a peripheral sensor of hypoxic burden (36, 37). We postulate that the upregulation of hemoglobin complex genes may acts as a compensatory mechanism to enhance oxygen-carrying capacity and mitigate cellular hypoxia induced by the bioenergetic deficits (38, 39) often seen in depression. This finding adds a new dimension to the pathophysiology of MDD, suggesting that the disorder involves systemic adaptations to metabolic demand beyond central nervous system signaling. Although PBMC preparations are generally depleted of erythrocytes, we cannot fully exclude subtle shifts in cell composition. However, the consistent upregulation across multiple erythroid-related genes and their association with clinical severity argue against a simple contamination effect.

In contrast to the upregulation of hypoxic and inflammatory markers (IL-6), we observed a significant downregulation of IGF1, MMP9, CSF2, and CXCR1. This “downregulated cluster” points towards a deficit in neurotrophic support and immune-mediated repair. IGF-1 is a potent neurotrophic factor essential for neurogenesis and synaptic plasticity (40); its peripheral reduction aligns with the “neurotrophic hypothesis” of depression, reflecting a deficit that may impair the maturation of stress-regulatory circuits during adolescence. However, peripheral IGF-1 levels are not consistently associated with depression in clinical populations (41–43), emphasizing the need to consider confounding factors such as disease duration, medication use, age, and whether the depression is first-episode or recurrent.

The observed discordance between IL-6 transcript and protein levels suggests the involvement of post-transcriptional regulatory mechanisms. IL-6 expression is tightly regulated at multiple levels, including mRNA stability, translational efficiency, and protein secretion, allowing for rapid and context-dependent immune responses. In peripheral immune cells, IL-6 mRNA can be transiently upregulated without a proportional increase in circulating protein levels, particularly under conditions of chronic or low-grade inflammation. Moreover, circulating IL-6 protein levels are influenced not only by cellular production but also by clearance dynamics and receptor-mediated consumption. Transcriptomic and proteomic measurements represent snapshots of distinct regulatory layers that may not be synchronized in cross-sectional designs. Therefore, the inverse or weak correspondence between IL-6 mRNA and protein observed in this study likely reflects complex regulatory processes rather than technical inconsistency.

Crucially, our partial correlation analysis (adjusted for age and gender) revealed that lower expression of MMP9 was significantly associated with more severe depressive symptoms (higher HAMD scores). Traditionally, elevated MMP9 is viewed as a marker of acute neuroinflammation and BBB disruption (44). However, MMP9 is pleiotropic, beyond its proteolytic activity in inflammation, it plays a constitutive and indispensable role in synaptic plasticity, specifically in the conversion of pro-BDNF to mBDNF and the maintenance of L-LTP (45, 46).

In our chronic cohort, the observed downregulation represents a “plasticity deficit” state rather than an active inflammatory state. The concurrent low levels of IGF-1 further suggest a failure of neurotrophic support. We propose a working model in which chronic depressive states may evolve from an initial phase dominated by inflammatory activation toward a subsequent plasticity-deficient state. Within this framework, reduced baseline levels of MMP9 and IGF1 may reflect an impaired capacity for synaptic remodeling and neuronal support. Consequently, the observed negative association between MMP9 expression and symptom severity may represent a “burnt-out” adaptive state, characterized by insufficient molecular resources to sustain synaptic plasticity and resilience, ultimately favoring neurotoxic dominance (47). Importantly, this model further suggests that therapeutic strategies aimed at restoring synaptic remodeling capacity may preferentially benefit patients exhibiting lower baseline MMP9 levels.

To translate these molecular signatures into clinical utility, we employed a multi-algorithm feature selection pipeline (LASSO, SVM-RFE, and Random Forest) to identify robust biomarkers, prioritizing stability despite the limited sample size. We identified a consensus diagnostic panel comprising SLC4A1, IGF1, and MMP9. This model achieved an AUC of 0.867, demonstrating that combining markers from distinct biological pathways yields a diagnostic fingerprint superior to single markers alone. Biologically, this three-gene signature suggests a coherent pathological trajectory: SLC4A1 captures the systemic physiological toll (metabolic/hypoxic stress); IGF1 signals the withdrawal of essential neurotrophic support; and MMP9 reflects the ultimate failure of the machinery required for synaptic adaptation and repair. Together, they describe a system under stress that has lost the capacity to remodel and recover. Although IGF1 demonstrated perfect classification in this dataset, this result should be interpreted conservatively, as small sample sizes are prone to overestimating predictive performance, even under cross-validation. Therefore, the observed accuracy likely represents an upper-bound estimate rather than true generalizability. The strength of IGF1 in this study lies in its biological consistency across transcriptomic and proteomic layers, rather than its apparent standalone diagnostic performance.

Several limitations of this study should be acknowledged. First, the sample size was relatively modest (N = 30). Although we employed strict statistical corrections, including Stratified Stability Selection and LOOCV, to minimize overfitting, validation in larger, multi-center cohorts is necessary to confirm the generalizability of the three-gene panel. Second, the cross-sectional design prevents causal inference; it remains to be determined whether the erythroid compensation is a driver of MDD or a downstream consequence of chronic physiological stress. Third, while PBMCs serve as a valuable window into systemic physiology, they may not fully recapitulate the region-specific synaptic alterations within the CNS.

## 5 Conclusion

In summary, our study delineates a systemic molecular signature of adolescent MDD defined by the coexistence of hypoxic compensation (SLC4A1 high) and neurotrophic/remodeling failure (IGF1/MMP9 low). We identified a robust three-gene candidate biosignature (SLC4A1, IGF1, MMP9) that demonstrates promising discrimination between patients and controls. Furthermore, the specific correlation of downregulated MMP9 and CXCR1 with increased symptom severity provides new insights into the link between immune-plasticity deficits and disease progression. These findings highlight the potential of targeting the immune-metabolic interface for future precision medicine approaches in adolescent depression.

## Data Availability

The datasets presented in this study can be found in online repositories. The names of the repository/repositories and accession number(s) can be found in the article/**Supplementary Material**.
