# Supplementary material for: Integrated transcriptomic and machine learning analysis reveals novel diagnostic biomarkers for adolescent major depressive disorder
Source: Front Psychiatry. 2026 Jan 30;17:1712225. doi: 10.3389/fpsyt.2026.1712225 (PMC12900750; doi:10.3389/fpsyt.2026.1712225)
Supplement: Supplementary file 1 [file DataSheet1.docx]

**Systemic Molecular Alterations in Adolescent Depression: Evidence from Blood-Based RNA Sequencing and Disease Enrichment Analyses**

Table S1. qPCR primer sequences of selected genes (5'→3')

| Gene | Primer | Sequence (5'→3') |
| --- | --- | --- |
| SLC4A1 | Forward primer | GGAGCTGGTGATGGACGAAA |
|  | Reverse primer | TCTATGCGGAACACCCTCTC |
| HBB | Forward primer | GGTGCCTTTAGTGATGGCCT |
|  | Reverse primer | CCCAGGAGCCTGAAGTTCTC |
| IGF1 | Forward primer | GACAGGCATCGTGGATGAGT |
|  | Reverse primer | ATGTACTTCCTTCTGGGTCTTGG |
| CSF2 | Forward primer | GCTGAGATGAATGAAACAGTAGAAG |
|  | Reverse primer | CCTTGGTCCCTCCAAGATGA |
| MMP9 | Forward primer | CAACTACGACACCGACGACC |
|  | Reverse primer | AATCGCCAGTACTTCCCATCCTT |
| CXCR1 | Forward primer | CTGGCCGGTGCTTCAGTTAGAT |
|  | Reverse primer | TTTGGCCGATGAAGGCGTAG |
| IL-6 | Forward primer | CCACCGGGAACGAAAGAGAA |
|  | Reverse primer | TCCTGGGGGTATTGTGGAGA |
| GYPA | Forward primer | GCCGACTGATAAAGAAAAGCCC |
|  | Reverse primer | TTCACCTGCATGTCCGGTTT |

Table S2. Information regarding the ELISA kits

| Reagent Name | Manufacturer | Catalog Number |
| --- | --- | --- |
| Human Insulin-like Growth Factor 1 Receptor (IGF1R) ELISA Kit | Enzyme-linked Biotechnology | MM-51454H1 |
| Human Granulocyte-Macrophage Colony Stimulating Factor (GM-CSF) ELISA Kit | Huamei Biological | CSB-E04568h |
| Human Matrix Metalloproteinase 9 (MMP-9) ELISA Kit | Bioswamp | HM10095 |
| Human CXC Chemokine Receptor 1 (CXCR1) ELISA Kit | Yaji Biological | YS01135B |
| Human Interleukin 6 (IL-6) ELISA Kit | Bioswamp | HM10205 |
| Human Band 3 Anion Transport Protein (SLC4A1) ELISA Kit | Tianjin Covino Biological Technology Co., Ltd. | KWN19265 |
| Human Hemoglobin Beta (HBb) ELISA Kit (High Sensitivity) | Shanghai Kpeirui Biological Technology Co., Ltd. | KPR-H12114 |
| Human Glycophorin A (GYPA) ELISA Kit | Shanghai Fusheng Industrial Co., Ltd. | A100610 |

| 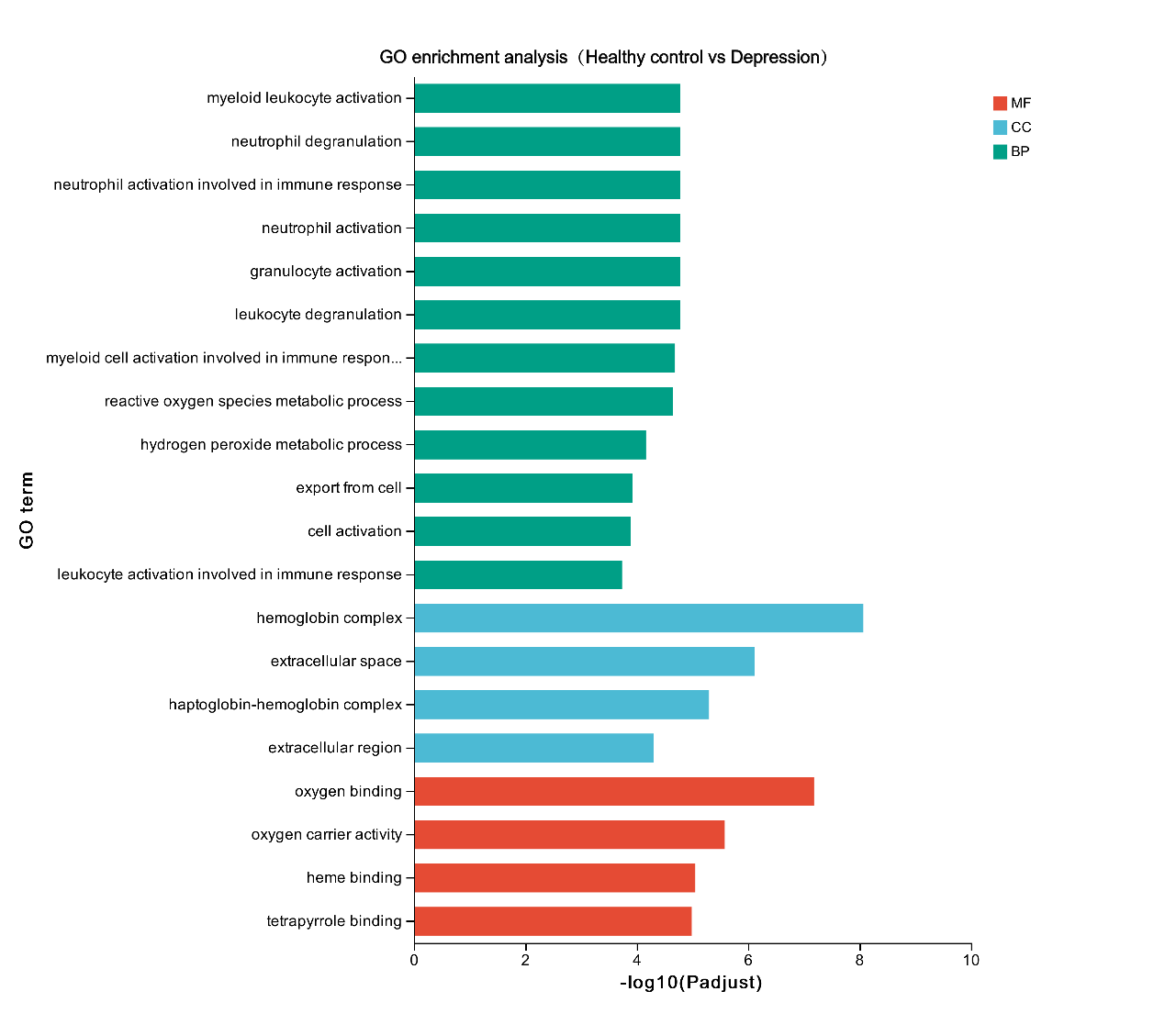 |
| --- |
| **Figure S1. Functional landscape of global transcriptomic alterations in adolescent MDD.** Gene Ontology (GO) enrichment analysis of the 367 nominally significant differentially expressed genes (DEGs). The bar plot illustrates the top enriched terms across three categories: Biological Process (BP, green), Cellular Component (CC, blue), and Molecular Function (MF, red). The x-axis represents the significance level (-log10 Adjusted P-value). The results highlight a dual signature characterized by the activation of innate immune responses (e.g., myeloid leukocyte activation) and compensatory metabolic processes (e.g., hemoglobin complex, oxygen carrier activity). |

| 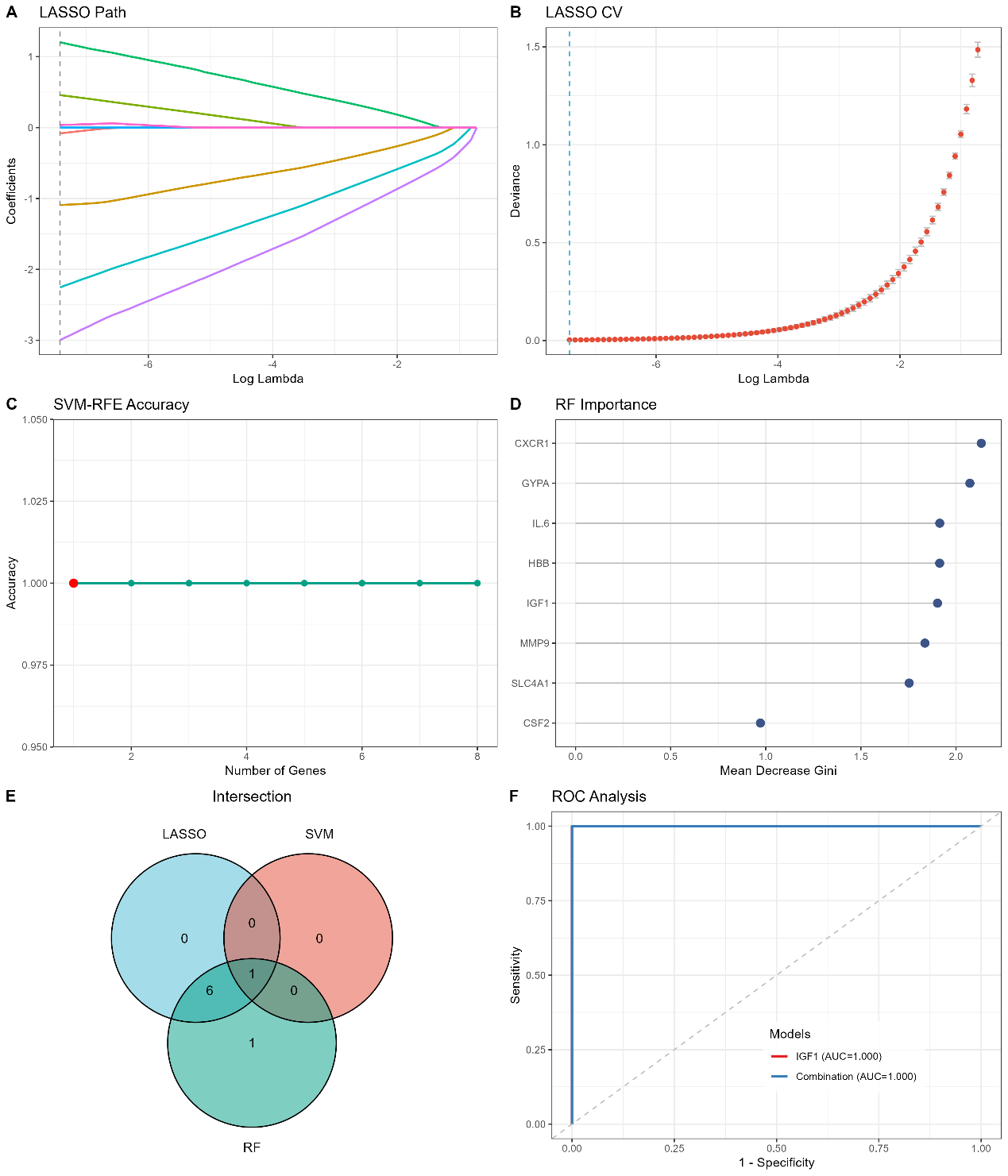 |
| --- |
| **Figure S2. Screening, identification, and validation of diagnostic biomarkers for MDD.** |

| 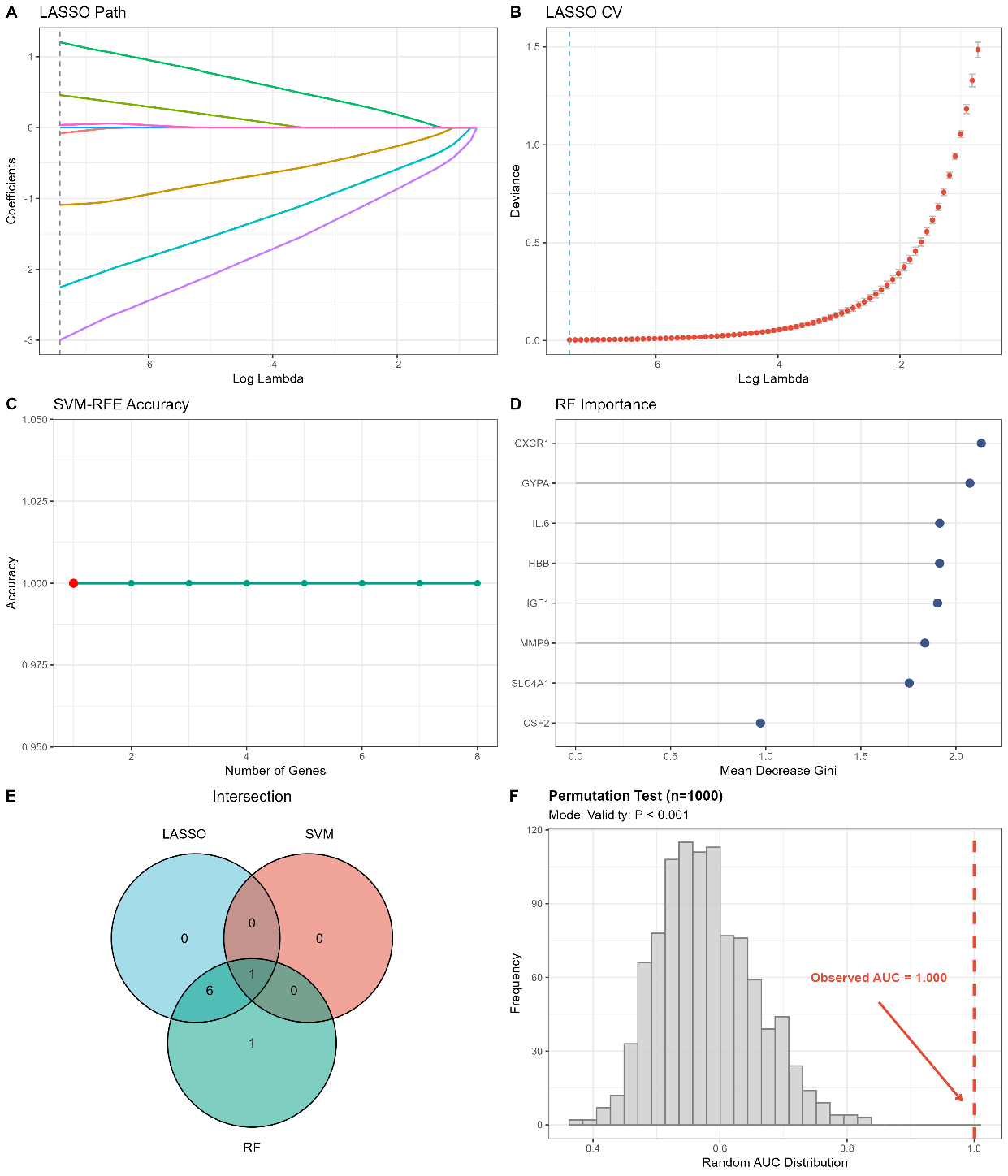 |
| --- |
| **Figure S3. Identification and validation of diagnostic biomarkers for MDD.** **(A-B)** LASSO regression analysis identified candidate biomarkers with non-zero coefficients. The cross-validation plot (B) shows minimal binomial deviance. **(C)** SVM-RFE analysis achieved 100% accuracy with a single feature, indicating a strong discriminative signal. **(D)** Random Forest importance ranking identified CXCR1, GYPA, and IL-6 as the top contributors to the classification. **(E)** Venn diagram illustrating the intersection of candidate genes identified by LASSO, SVM-RFE, and RF algorithms. One robust biomarker (*IGF1*) was shared by all three methods. **(F)** Permutation test (n=1,000) for model validation. The gray histogram represents the distribution of AUCs from models trained on randomly permuted labels, while the red dashed line marks the observed AUC of 1.000. The result (P < 0.001) confirms that the predictive performance is statistically significant and not due to overfitting. |

**Data availability**

The transcriptome data for this study are available at <https://ngdc.cncb.ac.cn/gsa-human/browse/HRA015120>.
